# Acupotomy by an ultrasound-guided technique: A protocol for a systematic review

**DOI:** 10.1097/MD.0000000000017398

**Published:** 2019-10-18

**Authors:** Zuyun Qiu, Yan Jia, Yifeng Shen, Qiaoyin Zhou, Xiaojie Sun, Xinyue Zhu, Shiliang Li

**Affiliations:** a Department of acupuncture-moxibustion, China-Japan Friendship Hospital, Beijing; b Chengdu University of Traditional Chinese Medicine, Chengdu, Sichuan; c Fujian University of Traditional Chinese Medicine, Fuzhou; d Beijing First Hospital of Integrated Chinese and Western Medicine, Department of Rehabilitation Medicine, Beijing, China.

**Keywords:** acupotomy, protocol, systematic review, ultrasound-guided technique

## Abstract

**Background:**

Acupotomy is a miniature surgery instrument. It can cut and detach the abnormal, cicatricial, and contractured tissues by causing only microtrauma. Acupotomy has been widely used clinically with a satisfactory efficacy. With the development of ultrasound technology, ultrasound-guided acupotomy has shown great value in clinical practice. But it is not yet clear that ultrasound-guided acupotomy is very effective and safe. Therefore, it is important to re-evaluate the available evidence to reach a relatively convincing conclusion that acupotomy by ultrasound-guided technique is a better choice than traditional acupotomy. The purpose of this systematic review is to provide a method for evaluating the effectiveness and safety of acupotomy by ultrasound-guided technique.

**Methods:**

This systematic review will be performed by searching relevant randomized controlled trials (RCTs) without any language or publication status restriction from inception to December 2019 by 2 researchers in nine databases (PubMed, Medline, Embase, Cochrane Library, Chinese literature databases, Chinese Biomedical Literature Database [CBM], China National Knowledge Infrastructure [CNKI], China Science and Journal Database [CSJD], and Wanfang Database). All RCTs evaluating acupotomy by the ultrasound-guided technique will be included in this study. Visual analog scale (VAS) and change of symptom will be assessed as the primary outcomes. The change in the ultrasound image, safety and adverse events, and acceptability will be assessed as secondary outcomes. The selection of study, data collection and analysis, and assessment of the study quality will be completed independently by 2 researchers. RevMan v.5.3 will be used for meta-analysis if no significant heterogeneity is detected. Continuous outcomes will be presented as the mean difference (MD) or standardized MD, while dichotomous data will be expressed as the relative risk.

**Results:**

This study will provide a high-quality synthesis of QL and AR to assess the effectiveness and safety of acupotomy by ultrasound-guided technique.

**Conclusion:**

This systematic review will provide evidence to judge whether acupotomy by ultrasound-guided technique is an effective the efficacy and safety intervention.

**PROSPERO registration number:**

CRD42018109070.

## 1 Introduction

Acupotomy is a miniature surgery instrument consisting of a handle, needle body, and blade.^[1]^ It can cut and detach the abnormal, cicatricial, and contractured tissues by causing only microtrauma.^[2]^ Acupotomy has been widely used clinically by doctors practicing traditional Chinese Medicine, orthopedics, and pain department in China with a satisfactory efficacy.^[3–6]^ Acupotomy is mainly used to treat chronic injuries of the motor system, cervical and lumbar diseases, and degenerative diseases of bones and joints, such as tenosynovitis, muscle injury, periarthritis of shoulder, cervical spondylosis, lumbar disc herniation, knee osteoarthritis, heel pain, etc. At the same time, some clinicians use it to treat internal medicine, gynecology, and dermatology-related diseases. At present, acupotomy practiced in the clinic is mostly in a traditional mode, where the whole operation process (only refers to the operation process) is guided by the experience and needling sensation of the operator and the subjective feeling of the patient, such as pain and numbness without the involvement of any modern medical aids tools.^[7]^ Therefore, it is impossible to completely avoid injuring some blood vessels and nerves or to affect the therapeutic effect because of the inaccurate location of the therapeutic target. Further, it is also not conducive to learning and transmission.

Acupotomy visualization is the inevitable direction of its future development.^[8]^ In the past, interventional diagnosis and treatment of musculoskeletal and articular diseases were usually carried out under the guidance of X-ray and CT.^[9]^ However, the display of soft tissue lesions in X-rays has greater limitations, and CT cannot observe the location of the acupotome tip in real-time. On the other hand, MRI is very tedious and expensive in assisting acupotomy in clinical practice.^[10]^ Because ultrasound has the advantages of fast, non-destructive, non-radiation emitting, and real-time monitoring as compared to X-ray, CT, and MRI, ultrasound-guided techniques have important auxiliary significance in clinical practice.^[11]^ With the development of ultrasound technology, ultrasound-guided technology has shown great value in clinical practice.^[12]^ Acupotomy by ultrasound-guided technique includes two methods: ultrasound localization and real-time monitoring. The advantages of acupotomy by ultrasound-guided lie in the following 2 aspects:

(1) Precise positioning: Ultrasound images can show the structural layers of the tissues clearly so that we can accurately identify the location of the lesions (muscles, tendons, ligaments, joints, cartilages, blood vessels, and nerves) and types (inflammation, degeneration, trauma, tumors, etc),
(2) Real-time dynamic ultrasound-guide: Ultrasound image can accurately display the position of acupotomy and its relationship with the adjacent tissues so as to have a good accuracy and safety. Some experiments have reported that acupotomy by ultrasound-guided has improved clinical efficacy and safety in cervical spondylosis,^[13–15]^ scapulohumeral periarthritis,^[16–18]^ lumbar disc herniation,^[19,20]^ knee osteoarthritis,^[21]^ and tenosynovitis,^[22,23]^ and it also reduces the incidence of adverse events.

There is no updated systematic evaluation or research program on this issue. It is also not yet clear that ultrasound-guided acupotomy is very effective and safe. Therefore, it is important to re-evaluate the available evidence to reach a relatively convincing conclusion that acupotomy by ultrasound-guided technique is a better choice than traditional acupotomy. This study will adapt the method of evidence-based medicine to analyze and evaluate clinical RCTs in acupotomy by an ultrasound-guided technique in order to provide evidence for further enhancing the clinical curative effects of acupotomy by ultrasound-guided technique. The study will assess the effectiveness and safety of the acupotomy using the ultrasound-guided technique.

## 2 Methods

### 2.1 Inclusion criteria for study selection

#### 2.1.1 Types of studies

All RCTs (randomized controlled trial, RCT) evaluating acupotomy by the ultrasound-guided technique will be included without any restriction to publication status or language. Non-RCT and uncontrolled clinical trials will be excluded. Any study with a sample size of <10 people will also be excluded from this review.

#### 2.1.2 Types of patients

All patients undergoing acupotomy by the ultrasound-guided technique will be included in the trial. All eligible patients will not be restricted by disease, age, sex, race, education, or economic status. Patients who will be decided unsuitable for acupotomy by ultrasound-guided technique, such as patients with fracture and dislocation, space-occupying lesions, cardiovascular and cerebrovascular diseases. and other serious diseases will be excluded.

#### 2.1.3 Types of interventions

##### 2.1.3.1 Experimental interventions

The treatment group will be treated with acupotomy by ultrasound-guided technique (there is no limit on the needle materials, ultrasonic equipment, and course of treatment).

##### 2.1.3.2 Control interventions

The control group will adapt to routine acupotomy, acupuncture, ultrasound-guided drug injection therapy, and oral western medicine treatment. The trial of acupotomy by ultrasound-guided technique with another active therapy compared to the same therapy alone will also be evaluated. Studies comparing different acupotomy insertion sites or different forms of acupotomy will be excluded.

#### 2.1.4 Types of outcome measures

##### 2.1.4.1 Primary outcomes

The primary outcome will be the change in the ultrasound image and the improvement of symptoms and functions of patients. This will be assessed through visual analog score (VAS) and the change in the ultrasound image. The emphasis will be on the changes in echo morphology and intensity of muscle, fascia, muscle bond, nerve, and fat in the ultrasound image.

##### 2.1.4.2 Secondary outcomes

(1) Quality of life
(2) Safety: Will be assessed by the incidence and severity of adverse reactions (eg, pain or bleeding).
(3) Acceptability: Will be assessed by withdrawal from the trial.

### 2.2 Search methods for the identification of studies

#### 2.2.1 Search databases

The RCTs evaluating acupotomy by ultrasound-guided technique without any language or publication status restrictions will be searched in nine databases (PubMed, Medline, Embase, Cochrane Library, Chinese literature databases, Chinese Biomedical Literature Database [CBM], China National Knowledge Infrastructure [CNKI], China Science and Journal Database [CSJD], and Wanfang Database) from inception to December 2019 The search terms include “acupotome”, “acupotomy”, “needle-knife”, “needle knife”, “ultrasound-guided technique”, “ultrasonic guidance”, “clinical article”, “clinical study”, “clinical trial”, “controlled study”, “randomized controlled trials”, and “prospective study”. The Chinese translations of these search terms will be used in the Chinese databases. The detailed search strategies in the PubMed database are presented in Table 1.

**Table 1:**
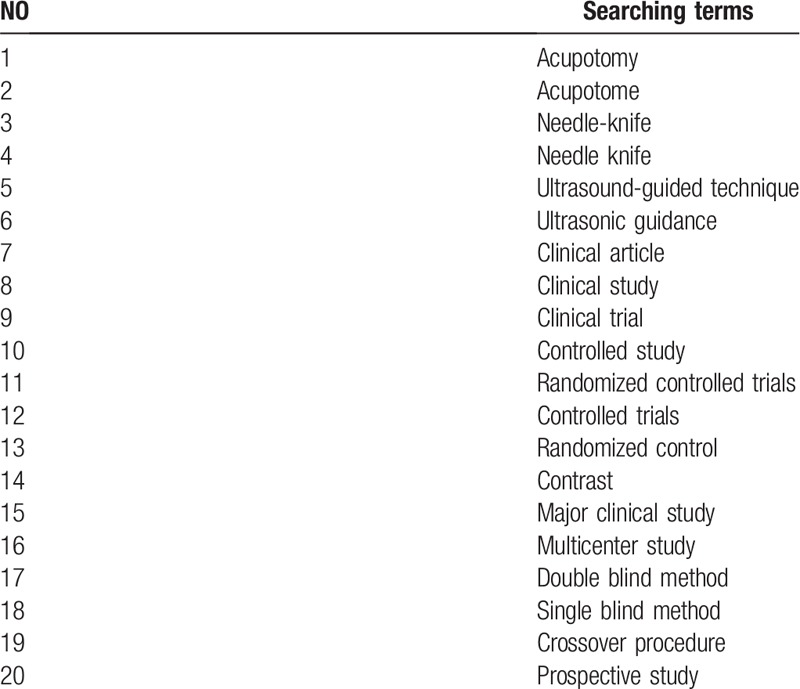
Details of the search strategy for PubMed.

#### 2.2.2 Searching other resources

The reference list of studies and systematic reviews related to acupotomy by the ultrasound-guided technique will be examined for additional trials. The relevant conference papers will be retrieved manually. The Clinical Trials.gov and the WHO International Clinical Trials Registry Platform (ICTRP) will be searched for new trials relevant to the topic.

### 2.3 Data collection and analysis

#### 2.3.1 Selection of studies

**Figure 1:**
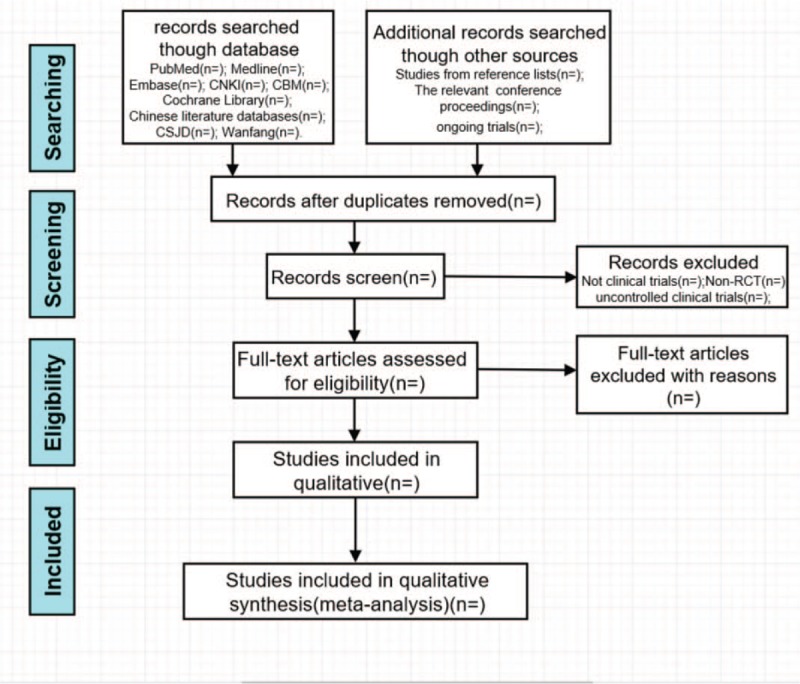
Process of research selection.

Researchers (ZQ and YJ) will import the retrieved literature into Endnote X7 and eliminate duplicate data. The noticeably below-standard articles will be excluded by 2 independent researchers (ZQ and YJ) after examining the titles and the abstracts. The researchers will then determine the preliminary inclusion of the literature by reading the full text, discussing in the group, and contacting the author for research details. Finally, the preliminary list will be cross-checked by 2 reviewers and another study member (SL) will resolve the inconsistencies and check the final literature that will be included. The final list of articles will be converted into Microsoft Excel format. The study-selection scheme of this systematic review is shown in Figure 1.

#### 2.3.2 Data extraction and management

Two researchers (ZQ and YJ) will extract and input data independently into the predefined data extraction form from all the selected articles’ data. Any discrepancies found during data cross-checking will be resolved by consensus or by the third reviewer (SL). The predefined data extraction form will include author information, publication time, participants, randomization, acupotomy by ultrasound-guided intervention, control intervention, indicators, research results, and adverse events. We will contact the investigator for further data, if necessary.

#### 2.3.3 Assessment of risk of bias in the included studies

Two researchers will use the Cochrane collaboration's tool to evaluate independently the risk of bias from seven dimensions: random sequence generation, allocation concealment, blinding method for patients, researchers and outcomes assessors, incomplete result data, and selective reporting. The risk of bias will be classified as low, unclear, and high.^[26]^ We will contact the investigator for further information if there is a lack of information on the risk of bias in the study. The assessment results will be cross-checked and disagreement will be resolved through discussion and arbitration with the third researcher (SL).

#### 2.3.4 Measures of treatment effects

The relative risk (RR) will be calculated to evaluate the enumeration data. The mean difference (MD) will be used to evaluate the measurement data. The effect sizes will be presented for analysis with a 95% confidence interval (CI).

#### 2.3.5 Unit of analysis issues

The unit of analysis will be the individual participants recruited into the trials.

#### 2.3.6 Dealing with missing data

We will attempt to contact the corresponding authors of the referenced articles to obtain the missing data. If the missing data cannot be obtained, we will perform our analysis based on the available data. If necessary, the potential impact of missing data on the final outcome of the review will be discussed.

#### 2.3.7 Assessment of heterogeneity

The heterogeneity of the results will be analyzed by the χ^2^ test (α = 0.1) and quantified by an *I*^2^ value. If *I*^2^ is ≤50%, the statistical heterogeneity among the trials can be negligible, and the effect size will be estimated using the fixed-effects model. If *I*^2^ is >50%, then there is significant heterogeneity among the trials.

#### 2.3.8 Assessment of reporting bias

When >10 trials are included in the study, funnel diagrams will be constructed to check the potential reporting bias. When the image is not clear, the software STATA 11.0 will be used to quantitatively analyze with the Egger test.

#### 2.3.9 Data synthesis

RevMan software (V.5.3) will be used for data synthesis. If substantial statistical heterogeneity is not detected in the results, the fixed-effects model will be employed for the meta-analysis. If there is substantial statistical heterogeneity, the source of the heterogeneity will be further analyzed. The random-effects model will be used for the meta-analysis after excluding the effects of obvious clinical heterogeneity. If there is significant clinical heterogeneity, subgroup, sensitivity analysis, or only descriptive analysis will be performed.

#### 2.3.10 Subgroup analysis exists

If substantial statistical heterogeneity exists in the included trials, we will conduct subgroup analysis according to the disease types and types of control interventions.

#### 2.3.11 Sensitivity analysis

If possible, we will perform sensitivity analysis to validate the robustness of the conclusions of the review according to the following:

(1) sample size,
(2) effects of missing data, and
(3) methodological quality.^[27]^

In addition, we will perform a repetitive analysis after excluding studies with low methodological quality.

#### 2.3.12 Assessment of the quality of evidence

We will evaluate the quality of evidence by the Grading of Recommendations Assessment, Development, and Evaluation (GRADE) and rate it according to very low, low, moderate, or high 4 levels.^[27,28]^

#### 2.3.13 Ethical principles and publication

It is not necessary to obtain ethical approval because this review does not involve personal information or impair the rights of the individuals. The results of this review will be published in peer-reviewed journals or conference reports.

## 3 Discussion

The purpose of this study is to evaluate the efficacy and safety of acupotomy by ultrasound-guided technique. The flow-chart of this systematic review is shown in Figure 1. The conclusions drawn from this review may benefit patients who are ready for acupotomy, as well as, clinicians, and decision makers. This review has some potential limitations. First, different types of needle knives, ultrasound equipment, and diseases may lead to heterogeneities. Second, the quality of the included studies may be poor, which will limit the ability to produce conclusions having high reliability. Third, the scope of literature sources is limited. Although there are studies evaluating acupotomy by the ultrasound-guided technique in the English language, most of the studies are reported in Chinese.

From the perspective of evidence-based medicine, acupotomy by ultrasound-guided technique is still controversial for the treatment of some diseases. Although studies have shown that ultrasound-guided technique can improve the efficacy and safety of acupotomy and reduce the occurrence of adverse events, these effects have not been evaluated scientifically and systematically. At present, there is no updated systematic evaluation or research undergoing on this issue, and it is not yet clear whether acupotomy by ultrasound-guided technique is effective and safe. Therefore, it is important to perform a systematic review system to reach a relatively convincing conclusion that ultrasound-guided technique is a better choice for acupotomy.

## Author contributions

**Conceptualization:** Yifeng Shen, Xinyue Zhu.

**Data curation:** Zuyun Qiu, Yan Jia.

**Formal analysis:** Yan Jia, Yifeng Shen, Qiaoyin Zhou, Xiaojie Sun, Xinyue Zhu.

**Investigation:** Zuyun Qiu, Yan Jia.

**Methodology:** Yan Jia, Yifeng Shen.

**Software:** Yifeng Shen, Qiaoyin Zhou, Xiaojie Sun, Xinyue Zhu.

**Supervision:** Shiliang Li.

**Validation:** Shiliang Li.

**Visualization:** Zuyun Qiu.

**Writing – original draft:** Zuyun Qiu, Yan Jia.

**Writing – review & editing:** Shiliang Li.
